# Acquired *ERBB2* Amplification and Overexpression as On-Target Resistance Mechanisms to Zongertinib With Subsequent Response to Trastuzumab-Deruxtecan: A Case Report

**DOI:** 10.1016/j.jtocrr.2026.100960

**Published:** 2026-01-22

**Authors:** David John McMahon, Alexius John, Foteini Kalofonou, Nawa Amin, Sarah Sarker, Joanna Vick, Nadia Yousaf, Nadza Tokaca, JanLukas Robertus, Suzanne MacMahon, Sanjay Popat

**Affiliations:** a Lung Unit, Royal Marsden Hospital, London, United Kingdom; b Lung Unit, Royal Marsden Hospital, Sutton, United Kingdom; c Histopathology Department, Royal Brompton Hospital, London, United Kingdom; d Molecular Diagnostics Department, Royal Marsden Hospital, Sutton, United Kingdom

**Keywords:** ERBB2, HER2, Case report, Zongertinib, T-DXd

## Abstract

A number of drugs are in development for the treatment of *ERBB2*(*HER2*)-mutated NSCLC, including antibody-drug conjugates such as trastuzumab-deruxtecan and tyrosine kinase inhibitors such as zongertinib and sevabertinib. Herein, we report a case of relapsed advanced *ERBB2*-mutant NSCLC with acquired resistance to zongertinib potentially mediated through *ERBB2* amplification and *HER2* 3+ immunohistochemistry overexpression with subsequent durable response to fifth-line trastuzumab-deruxtecan. We propose this as a mechanism for zongertinib resistance, one that may underpin a biological rationale for future *ERBB2* tyrosine kinase inhibitor–antibody-drug conjugate combination therapy.

## Introduction

*ERBB2* plays an important role in the regulation of cell survival and proliferation.^1^ *ERBB2* aberrations include protein overexpression, gene amplification, and genetic mutations. These aberrations can be present alone or in combination and have varying sensitivity to *ERBB2*-directed therapies. In NSCLC, *ERBB2* mutation and amplification are generally mutually exclusive.^2^^,^^3^ In NSCLC, *ERBB2* mutations (*ERBB2*m) are key markers of benefit with targeted treatment. Trastuzumab-deruxtecan (T-DXd), an *ERBB2*-directed antibody-drug conjugate (ADC), is approved by the Food and Drug Administration (FDA) and European Medicines Agency to treat *ERBB2*m NSCLC, based on the DESTINY-Lung02 trial.^3^ This demonstrated an objective response rate (ORR) of 49% to 56% and a median duration of response of 16.8 months and not estimable at two dose levels. A first-line phase 3 trial of T-DXd in *ERBB2*m NSCLC is ongoing.

T-DXd also has a tumor-agnostic FDA approval for the treatment of *ERBB2* for expressing solid tumors, based on the DESTINY-PanTumor01 and DESTINY-Lung01 trials.^4^ with blinded independent central review–confirmed ORR of 26.5% to 34.1% at two dose levels in the second-line setting.^5^

Two *ERBB2*-targeting tyrosine kinase inhibitors (TKIs) are in late-stage development.

Zongertinib is an irreversible TKI directed against *ERBB2*, which spares *EGFR*, and has demonstrated activity in *ERBB2*m NSCLC, with FDA breakthrough therapy designation.^1^ This is based on the BEAMION-Lung01 trial, which demonstrated an ORR of 71% and median progression-free survival of 12.4 months in a pretreated cohort. Priority review has also been granted by the FDA for BAY2927088 (sevabertinib), a reversible TKI, which has an ORR of 59.3% in a pretreated cohort.^6^ First-line phase 3 trials of these two TKIs are ongoing.^7^^,^^8^

There are also multiple other ADCs and TKIs in development. The optimal sequencing of these TKIs, ADCs, and current standard therapies is yet to be elucidated because there are little data to guide this. ADC and TKI sequencing may prove to play a major role in *ERBB2*m NSCLC therapeutics, but optimal sequence strategy (if any) remains unknown. Alongside this, resistance mechanisms to zongertinib and sevabertinib are not yet characterized and may assist in sequencing treatment decisions or even decisions for TKI-ADC combinations because of potential for synergy.

Here, we present a case of response to fifth-line T-DXd in advanced NSCLC with putative *ERBB2* amplification and overexpression as mechanism of resistance to immediate prior zongertinib.

## Informed Consent

The patient has provided informed consent for the publication of this manuscript.

## Case Presentation

In March 2021, a 62-year-old White man with no prior tobacco exposure was diagnosed with having *ERBB2* (Y772_A775dup)-mutant NSCLC adenocarcinoma and underwent resection. The final histology was pT2aN0PL1R0. PD-L1 tumour proportion score was more than 50%. *ERBB2* status was not tested using immunohistochemistry (IHC). Tissue DNA and RNA identified an *ERBB2*m (Y772_A775dup) but no *ERBB2* amplification nor copy number gain.

In March 2023, the patient relapsed with bone and mediastinal disease. Endobronchial ultrasound of stations 7 and 11L confirmed TTF-1–positive adenocarcinoma. Next-generation sequencing (NGS) was unchanged, detecting the parent *ERBB2* mutation with no *ERBB2* copy number gain. *ERBB2* IHC was 1+ (gastric scoring). In May 2023, the patient developed brain metastases and spinal cord compression (C6–T3) requiring both palliative spinal radiotherapy (20 Gy in five fractions) and stereotactic radiosurgery to seven brain metastases. He commenced first-line carboplatin-pemetrexed, remaining on maintenance pemetrexed until February 2024, when he began second-line weekly paclitaxel because of deteriorating performance status from liver, brain, pulmonary, and bony progressive disease. The patient also had multiple small cerebrovascular accidents during this time, because of progressive disease, and further palliative radiotherapy was delivered to the spine and pelvis (20 Gy in five fractions to lower spinal and pelvic disease).

In May 2024, the patient commenced compassionate-use third-line zongertinib with a brisk symptomatic improvement. Interval computed tomography (CT) after 5 weeks demonstrated partial response (reduction in size of pulmonary and hepatic metastases, approximately 50% shrinkage). This response was maintained for 8 months. CT in January 2025 demonstrated progression of disease in the liver. The patient elected to continue on zongertinib beyond progression because of ongoing clinical benefit. CT in February 2025 demonstrated further progression in the liver and bone. Repeat liver biopsy in February 2025 demonstrated TTF-1–positive adenocarcinoma without neuroendocrine differentiation and *ERBB2* overexpression (IHC 3+, gastric scoring). In-house tissue DNA NGS demonstrated the parent *ERBB2* mutation (*Y772_A775dup*) along with *ERBB2* amplification (copy number gain >8) and *CDK12* amplification. No fusions or other actionable findings were identified on RNA NGS. Confirmatory fluorescent in situ hybridization was not performed because of resource constraints. He received one cycle of carboplatin-gemcitabine bridging therapy. The patient developed a further cerebrovascular accident and developed further brain metastases.

**Figure 1:**
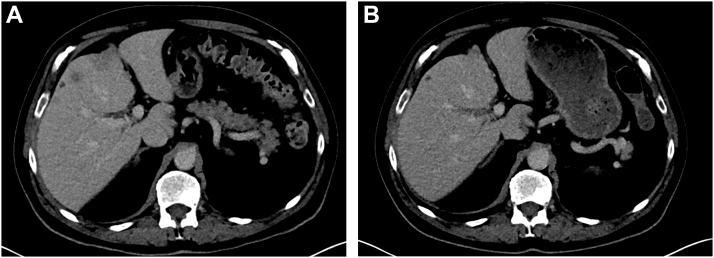
CT axial sections demonstrating response to therapy in liver and pleural disease between (*A*) cycle 1 day 1 T-DXd and (*B*) after 4 cycles. CT, computed tomography; T-DXd, trastuzumab-deruxtecan.

**Figure 2:**
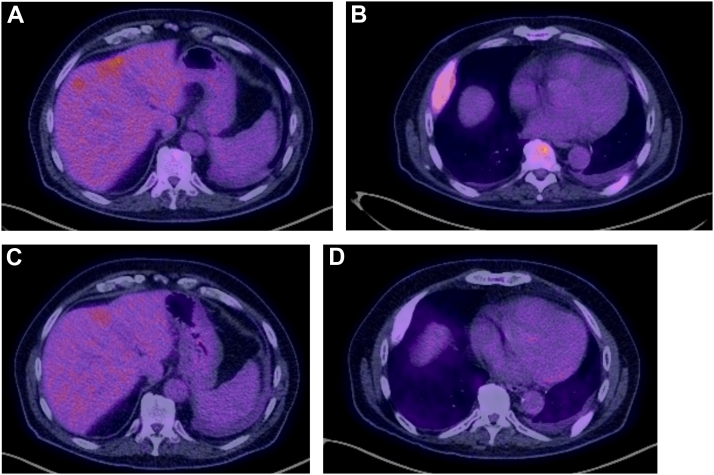
PET/CT axial sections demonstrating response to therapy in liver and pleural disease between (*A* and *B*) cycle 1 and (*C* and *D*) after 4 cycles of T-DXd. CT, computed tomography; PET, positron emission tomography; T-DXd, trastuzumab-deruxtecan.

In March 2025, the patient received the first cycle of T-DXd. In June 2025, after 4 cycles of T-DXd, CT and positron emission tomography/CT demonstrated response to therapy with reduction in size of liver lesions and reduction in SUV_max_ in bone, pleural, and liver metastases (Figs. 1 and 2), underpinned by a marked symptomatic improvement. This included reduction in fatigue, returning to work and hobbies, improved balance, and improved exercise tolerance. Treatment has been tolerated without evidence of significant toxicity to date, and response is ongoing while remaining on treatment in January 2026.

Restaging CT in November 2025 revealed maintained response (Response Evaluation Criteria in Solid Tumors version 1.1 partial response), and positron emission tomography/CT now reveals evidence of complete metabolic response. The patient continues on T-DXd with ongoing clinical benefit, after 8 months of T-DXd.

## Discussion

**Figure 3:**
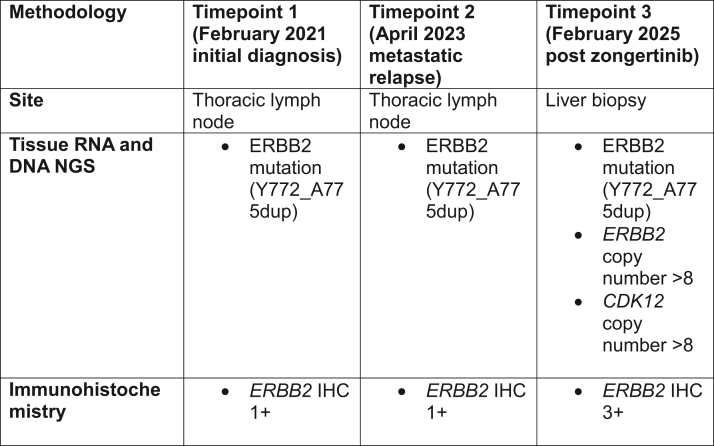
Timeline of histopathologic and molecular changes.

Treatment options for *ERBB2*m NSCLC are rapidly emerging, and this outpaces scientific advancements to guide sequencing of these therapies. The patient had significant clinical benefit with zongertinib for 8 months, before developing extracranial resistance. We identified co-occurring and related on-target resistance mechanisms to zongertinib, through both *ERBB2* amplification (detected through tissue NGS) and overexpression (detected through IHC) (Fig. 3). Whether these changes may have occurred because of clonal selection during prior chemotherapy or zongertinib is unclear, as tissue biomarker testing was not performed immediately before zongertinib treatment. However, given the mechanism of action of zongertinib, it is likely that these aberrations occurred on zongertinib and led to zongertinib resistance in this patient.

On-target mechanisms of resistance are common in other oncogene-addicted NSCLCs, for example, *EGFR*, *ALK*, and *ROS1*.^9^, ^10^, ^11^ Other mechanisms of resistance, such as histologic transformation or off-target mechanisms of resistance, for example, acquired *EGFR*, *MET*, or *ALK* aberrations, were not detected on this patient’s post-zongertinib biopsy. *ERBB2* overexpression and amplification have been identified as a common resistance mechanism to EGFR TKIs.^12^^,^^13^ Moreover, on-target copy number gain is well recognized as an acquired resistance mechanism to *EGFR* and *ALK* TKIs.^10^^,^^11^ Mechanisms of on-target resistance to zongertinib are yet to be determined in larger data sets, and, given its mechanism of action, are likely to consist of solvent front mutations, *C805X* gatekeeper mutations (where zongertinib binds), xDFG motif mutations, or compound mutations, none of which were identified in this patient.^14^^,^^15^

There are preclinical data and small numbers from prospective trials, indicating patients with either ERBB2 amplification or protein overexpression, or both in combination, may benefit from T-DXd; however, these models and patients had not previously been exposed to zongertinib.^3^^,^^16^ In a NSCLC context, T-DXd was demonstrated to have an ORR of 26.5% to 34.1% in two cohorts totaling 90 patients with *ERBB2*-overexpressing NSCLC without *ERBB2* mutation in the DESTINY-Lung01 trial, but this trial did not have an “*ERBB2*-low” cohort.^5^ Durable responses, including durable intra- and extra-cranial responses have been demonstrated in trials across many ERBB2 overexpressing cancers, most notably ERBB2-overexpressing breast cancer, leading to tissue-agnostic FDA accelerated approval for T-DXd.^4^^,^^17^^,^^18^

There are limited clinical data to guide physicians in sequencing the three most promising *ERBB2*-directed therapies (T-DXd, zongertinib, and sevabertinib). For example, there are no data we are aware of, describing response rates of patients to T-DXd after zongertinib, and this is the first published report of a response in this setting. This finding of complete metabolic response is a proof of concept, which requires validation in prospective studies and further real-world data sets, but it is mechanistically plausible. The reverse sequence of targeting *ERBB2*

(TKI after T-DXd) was evaluated in cohort 5 (N = 31) of the BEAMION LUNG-1 trial, demonstrating an ORR of 42% for zongertinib in patients who had previously received an *ERBB2-*directed ADC and ORR of 67% in patients naive to *ERBB2*-targeting drugs.^1^ A similar cohort in the SOHO-01 trial (expansion cohort E [N = 34]) demonstrated an ORR of 35% and median duration of response of 9.5 months with sevabertinib in patients pretreated with T-DXd.^6^ In addition, a case series of six patients pretreated with T-DXd and at least more than two other lines of therapy demonstrated a clinical benefit rate of 100% with zongertinib (complete response, N = 1; partial resposne, N = 4; SD, N = 1).^19^

Given this up-regulation of the *ERBB2* target we have identified through amplification and overexpression, we propose a mechanistic rationale for combination zongertinib with T-DXd, a combination with few overlapping toxicities, different mechanisms of action, and the potential for synergy.

## Conclusion

We report a case of a patient with *ERBB2*m NSCLC with progression on zongertinib, with post-zongertinib biopsies identifying *ERBB2* amplification and overexpression on tumor tissue as a potential resistance mechanism with subsequent durable response to T-DXd. Our case identifies the potential for the sequence of zongertinib followed by T-DXd and mechanistically proposes a rationale for combination therapy.

## CRediT Authorship Contribution Statement

**David John McMahon**: Conceptualization, Data curation, Formal analysis, Investigation, Methodology, Project administration, Validation, Visualization, Writing - original draft and final draft, Writing - review and editing.

**Alexius John**: Conceptualization, Writing - original draft and final draft review, Writing - editing.

**Foteini Kalofonou**: Writing - final draft, review, and editing.

**Nawa Amin**: Writing - final draft, review, and editing.

**Sarah Sarker**: Writing - final draft, review, and editing.

**Joanna Vick**: Writing - final draft, review, and editing.

**Nadia Yousa**f: Writing - final draft, review, and editing.

**Nadza Tokaca**: Writing - final draft, review, and editing.

**Robertus Jan Lukas**: Data curation.

**Suzanne MacMahon**: Data curation.

**Sanjay Popat**: Conceptualization, Project administration, Visualization, Writing - original draft and final draft, Writing - review and editing.

## Disclosure

Dr McMahon reports receiving travel assistance from Takeda. Dr John reports receiving honoraria from Boehringer Ingelheim and travel assistance from Takeda. Ms Vick reports serving as a speaker or receiving honoraria from Merck, Pfizer, and SBK Healthcare; receiving travel assistance from Merck Sharp & Dohme and Merck; having participation in data safety monitoring board of Johnson and Johnson; and receiving equipment/materials/other from Johnson and Johnson. Dr Tokaca reports providing consulting for Merck, Takeda, Pierre Fabre, and AstraZeneca; receiving honoraria/speaking fees from Merck, Takeda, Pierre Fabre, and AstraZeneca; and receiving travel support from Takeda and 10.13039/100004334Merck. Dr MacMahon reports serving as an assessor for GENQA/EMQN Prof Popat reports providing consulting and receiving personal fees from AnHeart Therapeutics, Amgen, Arcus Biosciences, AstraZeneca, Bayer, Bicycle Therapeutics, BioNTech, Bristol Myers Squibb, Boehringer Ingelheim, Daiichi Sankyo, Ellipses, Erasca, Genmab, Gilead, GlaxoSmithKline, Guardant Health, IO Biotech, Janssen/J&J, Lilly, Merck KGaA, Merck Sharp & Dohme, Neuvalent, Pfizer, Pharmamar, Pierre Fabre, Roche, Servier, Summit, Takeda, and Taiho; receiving honoraria from Amgen, AstraZeneca, Bayer, Gilead, Guardant Health, Janssen/J&J, Merck KGaA, PharmaMar, Pfizer, Roche, and Takeda; receiving travel support from 10.13039/100016016Gilead and 10.13039/100004337Roche; having participation in data safety monitoring board or advisory board: included in consulting fees previously mentioned; and having leadership role (not renumerated) from ALK Positive UK, Lung Cancer Europe, Ruth Strauss Foundation, BTOG Research Group, and ETOP-IBCSG Foundation Board. The remaining authors declare no conflict of interest.
